# Spectrum of malignant scalp tumours and its impact on management—a tertiary care cancer centre experience

**DOI:** 10.1186/s12957-023-03200-9

**Published:** 2023-10-17

**Authors:** Bipin T. Varghese, Abinaya R. Nadarajan, Shaji Thomas, Elizabeth Mathew Iype, Nebu Abraham George, Jagathnath Krishna K. M., Sahya S. Lal, Thara Somanathan

**Affiliations:** 1 Department of Head and Neck Oncology, Regional Cancer Center, Thiruvananthapuram, India; 2 Department of Surgical Oncology, Regional Cancer Center, Thiruvananthapuram, India; 3 Cancer Epidemiology & Biostatistics, Regional Cancer Center, Thiruvananthapuram, India; 4 Department of Pathology, Regional Cancer Center, Thiruvananthapuram, India

**Keywords:** Scalp tumour, Malignant, Reconstruction, Rotation flaps

## Abstract

**Background:**

Tumours on the scalp are diverse and often exhibit site- and histology-specific characteristics. Reconstructing the scalp after oncological resection has always been challenging because of its unique anatomy.

**Methodology:**

A retrospective review of patients with malignant scalp tumour operated on at a single institution over 10 years was performed. Data were collected and analysed regarding the scalp tumour profile, treatment, and the outcome of these procedures.

**Results:**

Of the 66 patients in our study, 33 (50%) had SCC. In addition to this, 21% were sarcomas, 17% were appendageal carcinomas, 11% were BCCs, and 1% was neuroendocrine carcinoma. Cortical erosion was observed in 6 patients in the CT imaging, all with SCC histology. Among the eight patients with pathological nodal involvement, three had angiosarcoma, three had SCC, one had appendageal carcinoma, and one had neuroendocrine carcinoma. The mean surgical defect size was 67.4 cm^2^. The surgical defect was reconstructed with local flaps in 58% of patients and primary closure in 27%. Local and systemic recurrence was noted in 25% of patients. Tumour size more than 6 cm, tumour histology (SCC & sarcoma), unplanned margin-positive excision, and residual disease in re-excision had higher recurrence, even though the *p*-value was not significant.

**Conclusion:**

Scalp tumours are heterogeneous in their clinical profiles. Often, its tumour biology and microscopic extent are underestimated. High suspicion, histological diagnosis, and clear surgical margins are all requirements in successfully treating scalp tumours. In order to minimize morbidity and restore an aesthetic and functional outcome, it is critical to use the simplest scalp reconstruction whenever possible.

## Introduction

A scalp has a unique anatomy because of its stratified structure. The scalp has five layers: the skin, connective tissue, galea aponeurotica, loose areolar connective tissue, and pericranium [1]. There are a large number of pilosebaceous follicles in the scalp that are surrounded by a dense network of capillaries and lymphatics [2]. Lymphatic drainage is oriented towards the occiput, pre- and postauricular regions, the upper neck, and the parotid glands. A total of 1–2% of scalp tumours are malignant, and they comprise up to 13% of all malignant cutaneous neoplasms [3]. Even though scalp neoplasms are generally benign, malignant ones deserve special attention due to their aggressive nature, high recurrence rates, and metastasis potential based on histologic subtype [4].

BCCs and SCCs are the most common primary malignant scalp tumours from the epithelium [2]. As a result of thick hair and the difficulty of self-inspecting certain areas, early detection is often difficult. A high index of suspicion based on the clinical presentation and the need for preoperative histological diagnosis are necessary before planning treatment. Imaging is essential to assess the extent of tumour invasion in the scalp, stage the disease, and plan treatment [4]. The spread of malignant scalp neoplasms begins with radial extension, with deep invasion commencing relatively late [5].

In the majority of cases, surgical excision is the standard of treatment. Histology plays a key role in determining the margins of resection and lymph node dissection, so it is always good to obtain a preoperative histological diagnosis. Reconstruction of the scalp after tumour removal requires more attention. Primary repair is used in small defects without tension; however, in large defects, local/regional flaps or free flaps are necessary [6].

## Materials and methods

We conducted a retrospective analysis on patients with malignant scalp tumours who underwent surgery at our institute in 10 years from January 2011 to January 2021. Data were obtained from the hospital’s electronic database and patient medical records. Patient demographic profile, clinical profile of scalp tumour, histological type, involvement of skull bone and nodes, the extent of resection, margin status, type of reconstruction, and recurrence were reviewed. The short-term clinical outcomes were evaluated. The minimum follow-up period was 24 months. This study was approved by the institutional review board and ethics committee. A waiver of informed consent was granted for the study, and all data were fully identified and anonymized before analysis. Statistical analysis was performed with SPSS software. The distributional properties of continuous variables were expressed as mean ± standard deviation, and categorical variables were expressed as frequency and percentage (%). Chi-square test and Fisher’s exact test were used to analyse the association between categorical variables. Logistic regression analysis was used to identify risk factors of recurrence.

## Results

There were 66 patients with histologically diagnosed malignant scalp tumours during the 10-year study period. Among these patients, 32 were men, and 34 were women. The mean age was 56 years. A total of 75% of the patients had an ulcerated lesion on the scalp and 25% with nodular lesions. The majority of the scalp lesions were located on the parietal (45%) and occipital regions (23%).

Out of 66 patients, 35 presented post-unplanned excision with margin involvement. Five patients with negative margins following excision at another centre had recurrence within 12 months. Re-excision showed residual disease in 28 of 35 patients (80%) post-margin-positive unplanned excision.

**Fig. 1:**
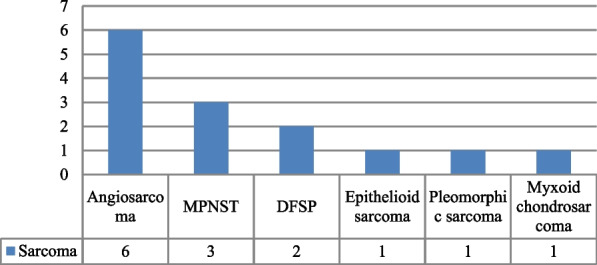
Histological spectrum of sarcoma in the study group. MPNST, malignant peripheral nerve sheath tumour; DFSP, dermatofibrosarcoma protuberans

Squamous cell carcinoma (SCC) was the most common histology noted in 33 patients (50%). Other histology observed included sarcoma (21%) (Fig. 1), appendageal carcinoma (17%), basal cell carcinoma (BCC) (11%), and neuroendocrine carcinoma (NEC) (1%). In four patients with SCC, the tumour was poorly differentiated. Among patients with SCC and sarcoma, 37% had pathological T1, 47% had T2, 11% had T3, and 5% had T4 disease. Pathological N1 disease was noted in 12% of the patients.

CT imaging revealed bone invasion in six patients. Among the 6 patients with bone invasion, all had squamous cell carcinoma. Final histopathology revealed bone involvement in only 3 out of 6 patients. Three of the six patients with suspected bone invasion in the preoperative imaging underwent outer table resection, while the others underwent craniectomy.

Ten patients had nodal involvement preoperatively based on the clinical and radiological evaluation. Seven patients had posterolateral neck dissections, 2 had parotidectomy for an intraparotid node, and 1 had parotidectomy with posterolateral neck dissection. According to the final histopathology report, 8 out of 10 patients had nodal involvement. There were nodal involvements in the following histology: SCC (3), angiosarcoma (3), appendageal carcinoma (1), and neuroendocrine carcinoma (1).

**Fig. 2:**
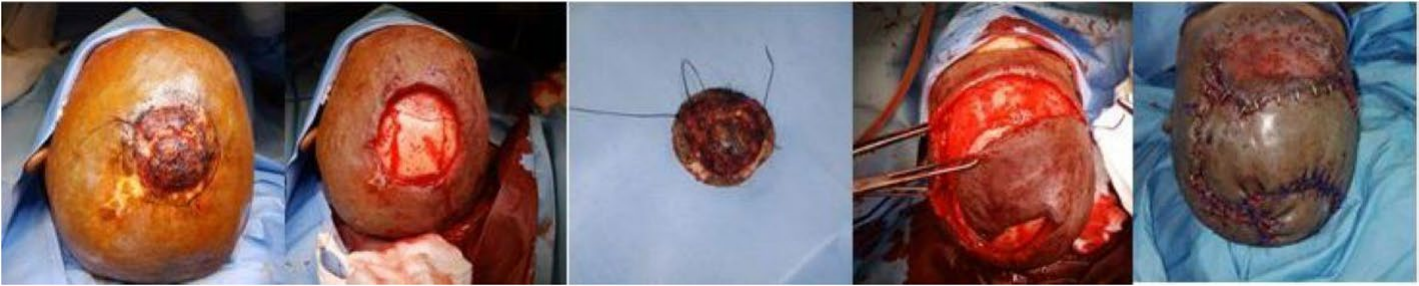
Tumour at vertex, post wide excision defect closed with rotation flap and SSG at donor site

**Fig. 3:**
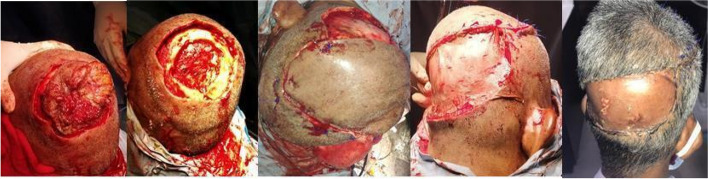
Wide excision with craniectomy without bone reconstruction. The defect covered with a rotation flap and SSG at the donor site

**Fig. 4:**
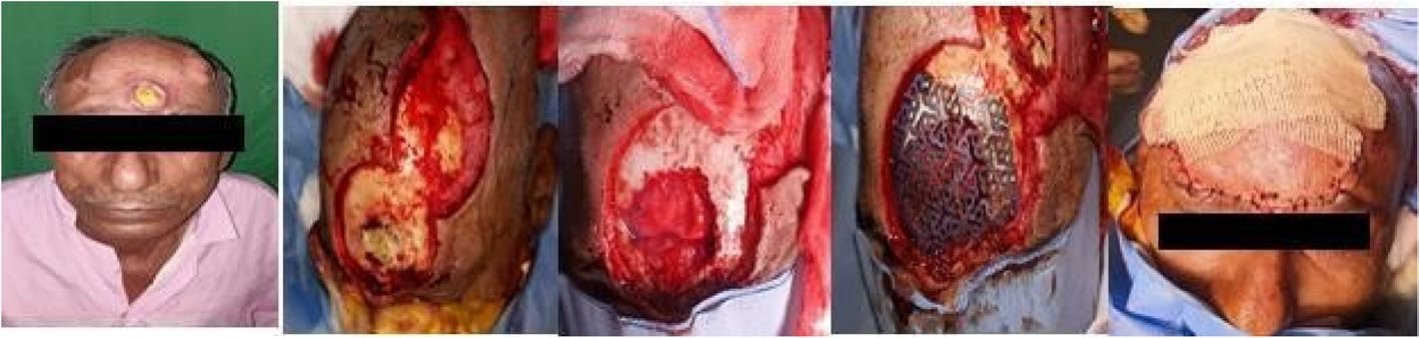
Frontal craniectomy defect reconstructed with titanium mesh and rotation flap

**Fig. 5:**
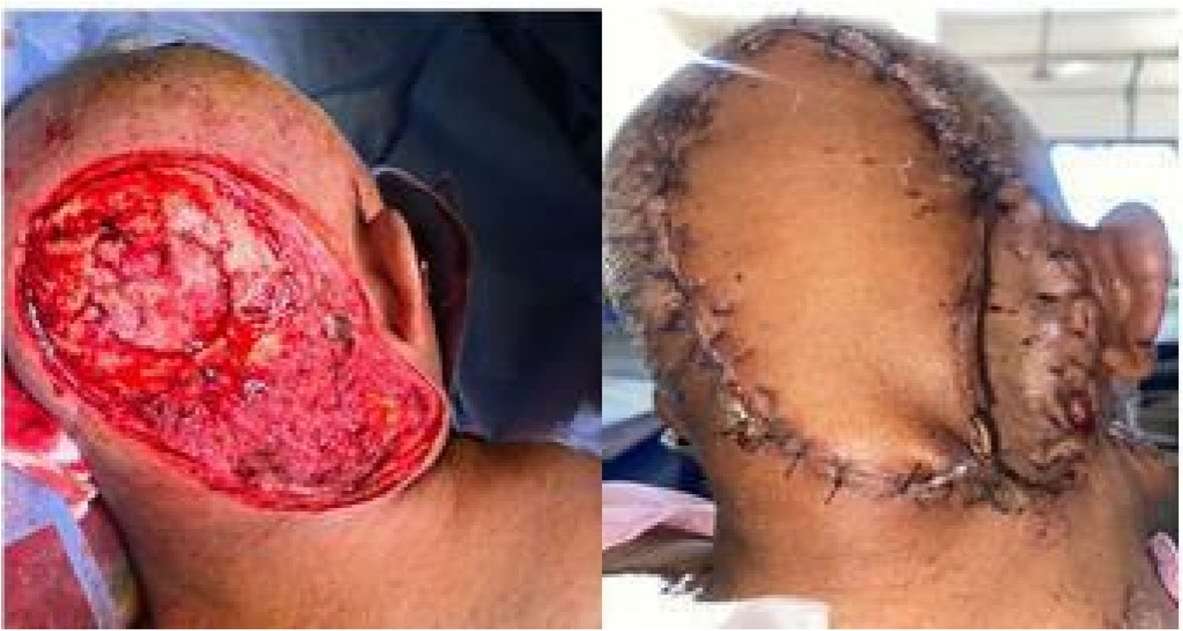
Post excision occipital craniectomy defect reconstructed with trapezius flap and SSG

**Fig. 6:**
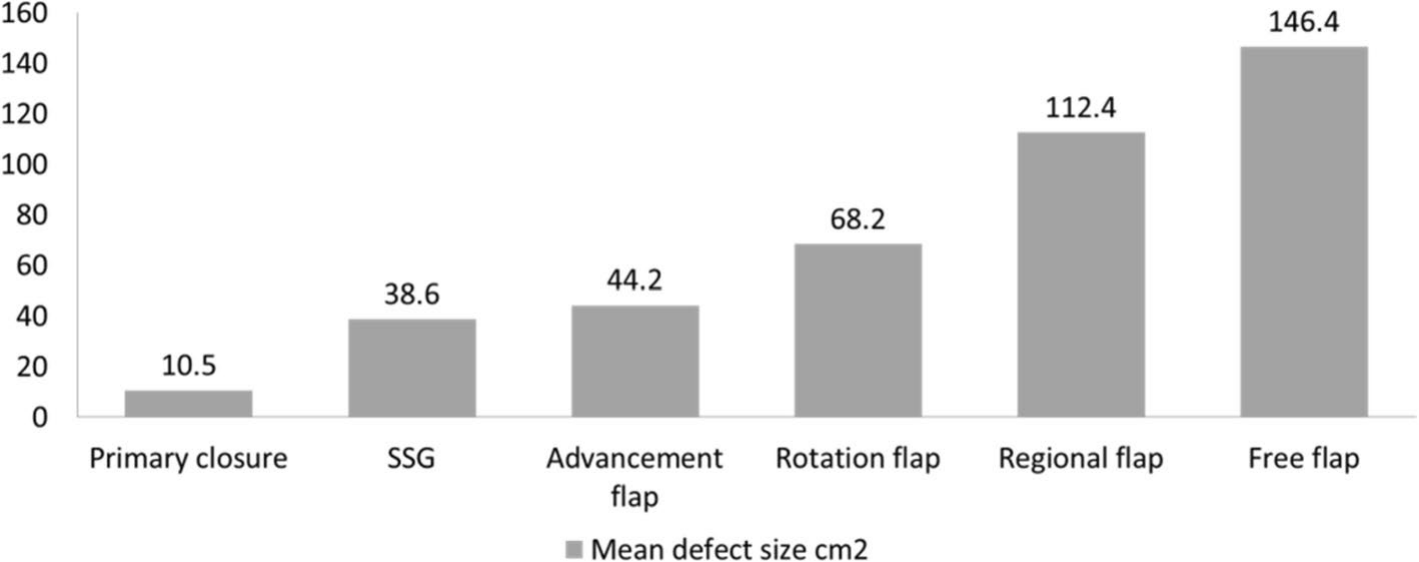
Mean defect size after excision for different reconstruction techniques

The mean defect size was 67.4 cm^2^, and rotation flaps (Fig. 2) were used most commonly (50%). In three patients with craniectomy defects, reconstruction was done with rotation flap (Fig. 3), trapezius flap, and titanium mesh reconstruction combined with rotation flap (Fig. 4). A regional flap reconstruction was performed on six patients: three with a trapezius flap (Fig. 5) and three with a temporal artery flap. Latissimus dorsi free flap reconstruction was performed in one patient with a large defect (Fig. 6).

In 12 patients, adjuvant radiation was used. Clinical follow-up was done every 3 months for the first 2 years, then every 6 months for the next 3 years, followed by an annual check-up. There were seventeen recurrences: 10 local recurrences, 3 nodal recurrences, and 4 systemic recurrences. Local and nodal recurrence was common in SCC, and systemic recurrence was common in sarcoma (Table 1). Univariate analysis suggested that variables such as age, gender, tumour site, size, histology, bone involvement, nodal involvement, unplanned margin positive excision, and residual disease in re-excision were not significantly associated with recurrence. Tumour size more than 6 cm, tumour histology (SCC & sarcoma), unplanned margin-positive excision, and residual disease in re-excision had higher recurrence, even though the *p*-value was not significant (Table 2).

**Table 1:** Clinical and treatment profile of various histological subtypes

Variables	SCC	Sarcoma	Appendageal carcinoma	BCC	NEC
Number	33	14	11	7	1
Mean age (years)	58	54	50	53	70
M/F	22/11	5/9	5/6	0/7	0/1
Site					
▪ Frontal	2	2	3	-	1
▪ Parietal	17	7	4	2	-
▪ Temporal	10	1	1	1	-
▪ Occipital	4	4	3	4	-
Defect reconstruction					
• Primary closure	5	5	6	2	-
• Rotation flap	19	8	4	2	-
• Advancement flap	3	-	-	2	-
• Regional flap	2 (trapezius)	1 (temp)	1 (trapezius)	1 (temp)	1 (temp)
• Free flap	1 (LD)	-	-	-	-
• SSG	3	-	-	-	-
• Nodal metastasis	3	3 (AS)	1	-	1
• Bone involvement	3	-	-	-	-
Recurrence					
• Local	6	2	2	-	-
• Nodal	2	1	-	-	-
• Systemic	-	4	-	-	-

Temp temporal artery-based flap, *LD* latissimus dorsi, *SSG* split-thickness skin grafting, *AS* angiosarcoma

**Table 2:** Binary logistic regression-risk factors for recurrence

Predictors for recurrence	OR (odds ratio)	95% CI for OR		Sig. (*p*-value)
		Lower	Upper	
Gender (male vs female)	0.786	0.26	2.375	0.67
Tumor size (> 6 vs < 6 cm)	**1.750**	0.173	17.686	0.635
Tumor histology	**1.957**	0.357	10.737	0.440
Bone invasion (yes vs no)	0.304	0.055	1.680	0.172
Nodal involvement (yes vs no)	0.542	0.137	2.147	0.383
Unplanned margin positive excision (yes vs no)	**1.488**	0.487	4.545	0.485
Residual disease in re-excision (yes vs no)	**1.173**	0.206	6.694	0.857
Adjuvant treatment (yes vs no)	0.889	0.279	2.836	0.842

## Discussion

The most common malignancies in Western populations are cutaneous malignancies, particularly BCC and SCC [2, 7, 8]. The most common histology in this study was SCC, followed by sarcoma. Melanoma is one of the most commonly detected scalp malignancies in Western populations, but none was seen in this study group [8, 9]. We found that scalp tumours are more common in middle-aged adults than in older people, according to Western literature [7].

Due to the rarity of these tumours and the ease with which they can be excised, more than 50% are excised without preoperative diagnosis and planning. Re-excision may result in a large defect, which could pose a challenge to the surgical oncologist regarding reconstruction. Approximately, 80% of unplanned excisions had a residual disease in re-excisions not detected by imaging.

Stratified scalp structures significantly influence tumour growth patterns due to resistance to infiltration, as only 4.5% of patients in this study had bone infiltration. As a result of the vertical limitations created by the cranial bones, tumours typically grow horizontally.

For most histological subtype, surgical excision with clear margins remains the standard of care. Margin criteria for scalp tumours vary by histology, and there is no standard. In the study group, all lesions were excised with a margin of at least 5 mm to 1 cm. An excision was performed up to the pericranium when the tumour was confined to the skin and connective tissues. In cases of suspected bone involvement, the outer table was excised, and a craniectomy was performed if full-thickness bones were involved. Some histological subtypes recur regardless of an adequate margin. Bone invasion and nodal metastases are common features of SCC. The most common sarcoma in this study group was angiosarcoma of the scalp, which is more likely to recur systemically.

Due to the dearth of tissue and the possibility of increased tension on the wound edges, primary closure is usually used for small defects of size 3–9 cm^2^ [10, 11]. The scarcity of tissue in the scalp and increased tension can impair healing. A total of 24% of patients in this study had primary closure. For all unplanned excisions with positive margins, re-excision with a 2-cm margin was done in all directions including the pericranium. Wider margins produced larger defects that required flap reconstruction following rewide excision. Advancement and rotation flaps, which are standard options for scalp reconstruction, provide good aesthetic results and a sufficient amount of tissue to cover large defects. Free flaps are rarely required for large defects. Studies have suggested that skin grafting, pedicled flaps, and free flaps are useful in reconstructing scalp defects larger than 30 cm^2^, 50 cm^2^, and 90 cm^2^, respectively [12, 13]. A mean defect size of 67.4 cm^2^ was observed in this study, and most defects were managed by local flaps.

It is crucial to plan meticulously for scalp reconstruction in order to proceed with wide excision without compromising oncological safety. Carefully planned rotation flaps can serve to manage small to complex defects even involving the underlying bone with or without concomitant bone reconstruction for which it is imperative to have a good understanding of anatomy and expertise in reconstruction. Figure 3 shows a patient who underwent a craniectomy without bone reconstruction and a defect covered only by a rotation flap and recovered well without compromising cosmetic or functional outcomes.

Our study has certain limitations. The data was retrospective, so we could not consider other parameters for analysis, such as exact margins given for different histology and other high-risk histological features. In view of small sample size, *p*-value was not significant for the risk factors to predict recurrence.

## Conclusion

Scalp malignancies are rare entities with a broad histological spectrum. Often, its biology and microscopic extent are underestimated. It is mandatory to have a high index of suspicion, appropriate imaging, a histological diagnosis, and proper surgical planning before performing surgical excision. Using the most straightforward reconstruction whenever possible is essential to reduce morbidity and provide a more functional and aesthetic outcome.

## Data Availability

The data analyzed during the current study are available from the corresponding author upon reasonable request.
